# GE Covariance Through Phenotype to Environment Transmission: An Assessment in Longitudinal Twin Data and Application to Childhood Anxiety

**DOI:** 10.1007/s10519-014-9659-5

**Published:** 2014-05-01

**Authors:** Conor V. Dolan, Johanna M. de Kort, Toos C. E. M. van Beijsterveldt, Meike Bartels, Dorret I. Boomsma

**Affiliations:** Department of Biological Psychology, FPP, VU University Amsterdam, Amsterdam, The Netherlands

**Keywords:** Simplex model, GE-covariance, Twin design, Phenotype-to-E transmission, Niche picking, Childhood anxiety

## Abstract

We considered identification of phenotype (at occasion t) to environment (at occasion t + 1) transmission in longitudinal model comprising genetic, common and unique environmental simplex models (autoregressions). This type of transmission, which gives rise to genotype-environment covariance, is considered to be important in developmental psychology. Having established identifying constraints, we addressed the issue of statistical power to detect such transmission given a limited set of parameter values. The power is very poor in the ACE simplex, but is good in the AE model. We investigated misspecification, and found that fitting the standard ACE simplex to covariance matrices generated by an AE simplex with phenotype to E transmission produces the particular result of a rank 1 C (common environment) covariance matrix with positive transmission, and a rank 1 D (dominance) matrix given negative transmission. We applied the models to mother ratings of anxiety in female twins (aged 3, 7, 10, and 12 years), and obtained support for the positive effect of one twin’s phenotype on the other twin’s environment.

## Introduction

The aim of this paper is to explore how the phenotype of children or adults may influence their own and their family members’ environment. We consider this in the context of the genetic simplex model as applied to longitudinal twin data. The genetic simplex model was proposed to investigate the covariance structure of longitudinal or repeated phenotypic measures in the classical twin design (Eaves et al. 1986; Boomsma and Molenaar 1987a). This involves fitting first order autoregressive models to the additive genetic (A), shared (or common; C), and unshared (or specific; E) covariance matrices of the repeated measures. Usually in its application, the variables A, C, and E are assumed to be uncorrelated, and to contribute additively to the variance of a continuous phenotype, or a liability underlying a discrete phenotype. We denote these assumptions as the absent of ‘GE covariance’ and ‘GxE interaction’, respectively. Note that the G and the E in this shorthand refer to genetic influences (A and/or D (dominance effects)) and environmental effects (unique and common) in general.

The inclusion of GE covariance in the genetic simplex model can be achieved in different ways. We are interested in the process in which the phenotype contributes to the environment (henceforth, Ph->E transmission). We limit ourselves to the effects of the phenotypes of the twins at occasion t on their environmental variables (E) at occasion t + 1 in the twin model, whether the transmission is model within and between twin members. This extension is inspired by sibling interaction models (Eaves 1976; Eaves et al. 1977; Carey 1986), which include mutual effects of the phenotypes of the twins and siblings on each other, and by an extension to the genetic simplex presented by Eaves et al. (‘phenotype-to-phenotype transmission’). We presented a related extension in de Kort et al. (2012), which, as we explain below, is a special case of the present model.

This extension can also be related to the processes of active and passive GE-covariance as discussed by Plomin et al. (1977) and (Scarr and McCartney 1983), and to the process of ‘genotypes selecting environments’ as discussed Eaves et al. (1977). Such processes, and so the GE-covariance arising from them, are considered to be plausible in cognitive development (Dickens and Flynn 2001; Johnson et al. 2011; Haworth et al. 2010), and in developmental psychopathology (Rutter et al. 1997; Rutter et al. 2006; Rutter and Silberg 2002). Given that most twin models do not include explicitly GE-covariance, the present paper may help to bring the practice of longitudinal twin modeling closer to the theory of developmental psychology.

The immediate goal of our paper is to present the phenotype to environment transmission model, to establish that the modeling including Ph->E transmission in the standard genetic simplex model is locally identified, and that the model is empirically viable in terms of resolution and statistical power. We consider identification in given 3 or 4 measurement occasions in the classical twin design, we apply the model to data obtained at 4 occasions.

Below we first present the standard simplex model. We then consider the extension consisting of the path from the phenotype to the unshared environmental influences within and between twin members. Local identification of the models is considered for 3 and 4 occasions analytically. We consider the issue of power to detect the effects associated with our extension, and we consider constraints, which may enhance the power. We apply the models to maternal ratings of anxiety in female twins at ages 3, 7, 10, and 12 years.

## The Standard Genetic Simplex Model

**Fig. 1:**
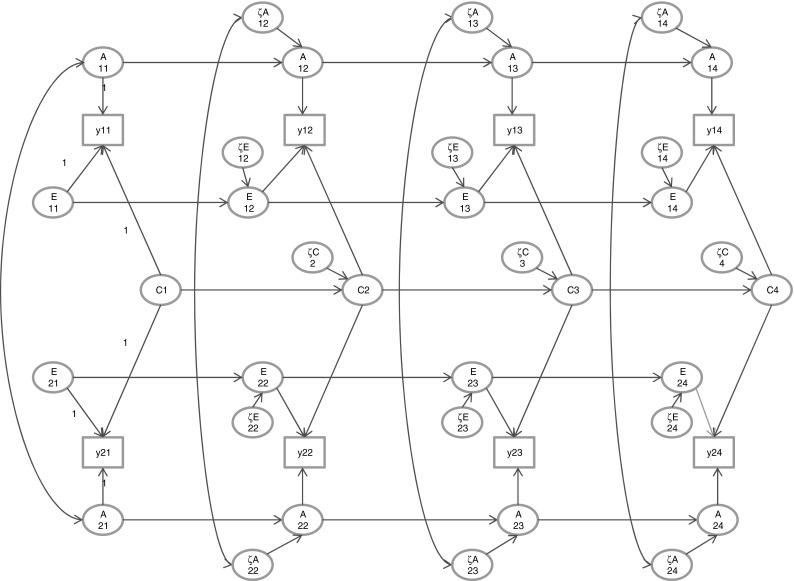
The standard ACE simplex (ACE model). Occasion-specific influences are not shown. The scaling used is shown only at t = 1

Let y_ijt_ denote the phenotypic score of member j (j = 1,2) of twin or sibling pair i at time or age t (t = 1,…,T; where T is 3 or 4). The phenotypic score is regressed on the A, C, and E variables: ${\text{y}}_{\text{ijt}} = {\text{b}}_{{0{\text{t}}}} + {\text{A}}_{\text{ijt}} + {\text{C}}_{\text{ijt}} + {\text{E}}_{\text{ijt}} + \varepsilon_{\text{ijt}}$, where all regressors have zero means, so that the phenotypic mean E[y_ijt_] equals the intercept b_0t_ (see Dolan et al. 1991, for a version with structured means). The occasion-specific residual is subject to the decomposition $\varepsilon_{\text{ijt}} = {\text{a}}_{\text{ijt}} + {\text{c}}_{\text{ijt}} + {\text{e}}_{\text{ijt}}$, where e_ijt_ possibly includes measurement error. The A, C, and E variables are subject to first order autoregressions (t = 2,…,T):

$$ {\text{A}}_{\text{ijt}} = {\text{b}}_{{{\text{At}},{\text{t}} - 1}} {\text{A}}_{{{\text{ijt}} - 1}} + \zeta_{\text{Aijt}} , $$

$$ {\text{C}}_{\text{ijt}} = {\text{b}}_{{{\text{Ct}},{\text{t}} - 1}} {\text{C}}_{{{\text{ijt}} - 1}} + \zeta_{\text{Cijt}} , $$

$$ {\text{E}}_{\text{ijt}} = {\text{b}}_{{{\text{Et}},{\text{t}} - 1}} {\text{E}}_{{{\text{ijt}} - 1}} + \zeta_{\text{Eijt}} , $$

where b_At,t−1_, b_Ct,t−1_, and b_Et,t−1_ are the autoregressive coefficients, and ζ_Aijt_, ζ_Cijt_, and ζ_Eijt_ are regression residuals (a.k.a, innovations in this context). At t = 1, we set A_ij1_ = ζ_Aij1_, C_ij1_ = ζ_Cij1_, and E_ij1_ = ζ_Eij1_. The model is depicted in Fig. 1. Identification of the standard genetic simplex is not an issue, as it is based on the decomposition of the phenotypic TxT covariance into the genetic and environmental (A, C and E) covariance matrices. This decomposition poses no problems of identification in the classical twin design and in other genetically informative designs. In simultaneously subjecting these covariance matrices to the simplex model, the standard identification conditions hold (Jöreskog 1970). Notably, given that the autoregressive parameters (b_At,t−1_, b_At,t−1_, b_At,t−1_) are not zero and the variances (σ^2^[ζ_At_], σ^2^[ζ_Ct_], σ^2^[ζ_Et_]) are not zero, the occasion-specific variances, σ^2^[a_t_], σ^2^[c_t_], and σ^2^[e_t_], are not identified at t = 1 and t = T. This is usually solved by fixing these to zero (e.g., σ^2^[a_1_] = σ^2^[a_T_] = 0; same applies to the environmental occasion-specific variances), or by equating the variance components at occasions 1 and 2, and at occasions T − 1 and T (e.g., σ^2^[a_t_] = σ^2^[a_t+1_], where t = 1 or t = T − 1; same applies to the environmental occasion-specific variances). Assuming identification is achieved by applying such constraints, the associated decomposition of variance is

$$ \sigma^{ 2} [{\text{y}}_{\text{ijt}} ] = \sigma^{ 2} [{\text{A}}_{\text{t}} ] + \sigma^{ 2} [{\text{C}}_{\text{t}} ] + \sigma^{ 2} [{\text{E}}_{\text{t}} ] + \sigma^{ 2} [\varepsilon_{\text{t}} ],\quad \left( {{\text{t}} = 1,{\text{T}}} \right) $$

$$ \sigma^{ 2} [\varepsilon_{\text{t}} ] = \sigma^{ 2} [{\text{a}}_{\text{t}} ] + \sigma^{ 2} \left[ {{\text{c}}_{\text{t}} } \right] + \sigma^{ 2} [{\text{e}}_{\text{t}} ], \quad \left( {{\text{t}} = 1,{\text{T}}} \right) $$

$$ \sigma^{ 2} [{\text{A}}_{\text{t}} ] = {\text{b}}_{{{\text{At}},{\text{t}} - 1}}^{ 2} \sigma^{ 2} [{\text{A}}_{{{\text{t}} - 1}} ] + \sigma^{ 2} [\zeta_{\text{At}} ],\quad \left( {{\text{t}} = 2,{\text{T}}} \right) $$

$$ \sigma^{ 2} [{\text{C}}_{\text{t}} ] = {\text{b}}_{{{\text{Ct}},{\text{t}} - 1}}^{ 2} \sigma^{ 2} [{\text{C}}_{{{\text{t}} - 1}} ] + \sigma^{ 2} [\zeta_{\text{Ct}} ],\quad \left( {{\text{t}} = 2,{\text{T}}} \right) $$

$$ \sigma^{ 2} [{\text{E}}_{\text{t}} ] = {\text{b}}_{{{\text{Et}},{\text{t}} - 1}}^{ 2} \sigma^{ 2} [{\text{E}}_{{{\text{t}} - 1}} ] + \sigma^{ 2} [\zeta_{\text{Et}} ].\quad \left( {{\text{t}} = 2,{\text{T}}} \right) $$

At t = 1, we set $\sigma^{ 2} [{\text{A}}_{ 1} ] = \sigma^{ 2} [\zeta_{\text{A1}} ],\sigma^{ 2} [{\text{C}}_{ 1} ] = \sigma^{ 2} [\zeta_{\text{C1}} ]$, and $\sigma^{ 2} [{\text{E}}_{ 1} ] = \sigma^{ 2} [\zeta_{\text{E1}} ]$ (given ${\text{A}}_{\text{ij1}} = \zeta_{\text{Aij1}} ,{\text{ C}}_{\text{ij1}} = \zeta_{\text{Cij1}} ,{\text{ and E}}_{\text{ij1}} = \zeta_{\text{Eij1}} )$.

The genetic simplex model and variations on this model (e.g.,Hewitt et al. 1988) have been put to good use in studies of personality, cognition, psychophysiology, psychopathology, etc. (e.g., see Hoekstra et al. 2007; Rietveld et al. 2003a, b; Bartels et al. 2004, 2002; Boomsma et al. 1989; Gillespie et al. 2007; Cardon et al. 1992; Petrill et al. 2004). Minica et al. (2010) discussed the inclusion of measured genetic variants in the genetic simplex. In these studies, GE-covariance was assumed to be absent. Below we extend the standard simplex model by considering the possibility that the phenotypes of the twins at occasion t contributes to their environmental influences at occasion t + 1. This extension introduces covariance among the A, C, and E, and necessarily alters the meaning of the latent environmental variables, as we point out below.

## Ph->E Transmission, in the Presence of C

**Fig. 2:**
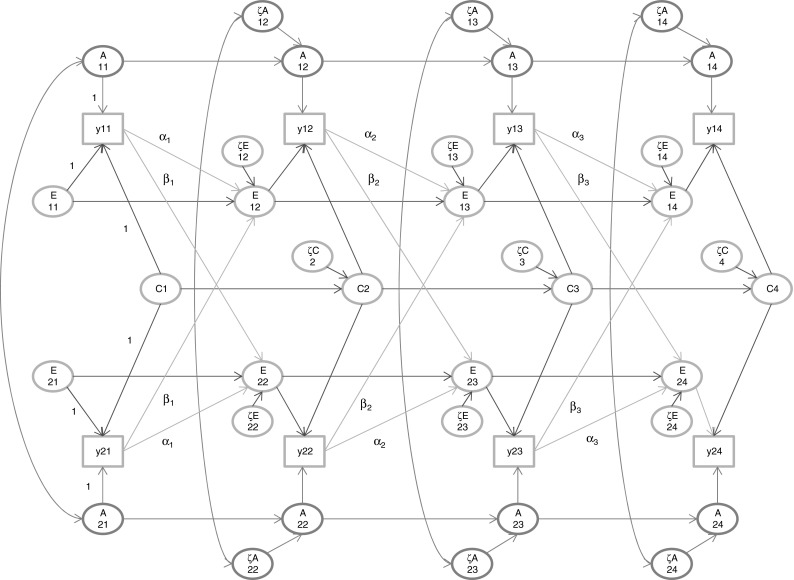
The extended ACE simplex model. Occasion-specific influences are not shown. The scaling used is shown only at t = 1. The extension comprises the arrows from the phenotype y^*^ at t to the E variables at t + 1 (i.e., parameters α_k_ and β_k_). For the distinction between y (Fig. 1) and y^*^ in this Figure, see the text

Figure 2 depicts the model in which we suppose that the phenotype of a person at occasion t contributes to shaping his or her own environment (say E_i1t+1_; parameters α_k_; k = 1,…,T − 1), and possibly also to the environment of a cotwin or other family members at occasion t + 1 (E_i2t+1_; parameters denoted β_k_; k = 1,…,T − 1). At t = 1, we assume that the environmental variables are not subject to such direct phenotypic influences, so that at t = 1 the latent environment variables have their standard interpretation, which in part is based on their specification as uncorrelated.

As above, the phenotype at each time point is related to the intercept b_0t_ (the phenotypic mean at time t) and the zero mean additive genetic (A_ijt_), environmental variables (C_ijt_ and E_ijt_), and the time specific residual (ε_ijt_):

$$ {\text{y}}_{\text{ijt}} = {\text{b}}_{{0{\text{t}}}} + {\text{y}}_{\text{ijt}}^{*} + \varepsilon_{\text{ijt}} , $$

$$ {\text{y}}_{\text{ijt}}^{*} = {\text{A}}_{\text{ijt}} + {\text{C}}_{\text{ijt}} + {\text{E}}_{\text{ijt}} . $$

The phenotype y_ijt_ is decomposed into a part, y_ijt_^*^, that is subject to the longitudinal model, and the time specific part, ${\text{b}}_{{0{\text{t}}}} + \varepsilon_{\text{ijt}}$, which, as above, may include zero mean occasion-specific influences: $\varepsilon_{\text{ijt}} = {\text{a}}_{\text{ijt}} + {\text{c}}_{\text{ijt}} + {\text{e}}_{\text{ijt}}$. In this manner, the time specific influences ε_ijt_ are strictly time specific, i.e., not subject to any transmission. As above, the additive genetic variable (A) and shared environmental variable (C) is subject to the first order autoregressions. The environmental variables in twin members 1 and 2 are regressed on the preceding environmental variables and preceding phenotypes:

$$ {\text{E}}_{\text{i1t}} = {\text{b}}_{{{\text{Et}},{\text{t}} - 1}} {\text{E}}_{{{\text{i1t}} - 1}} + \alpha_{\text{k}} {\text{y}}^{*}_{{{\text{i1t}} - 1}} + \beta_{\text{k}} {\text{y}}^{*}_{{{\text{i2t}} - 1}} + \zeta_{\text{Ei1t}} , $$

$$ {\text{E}}_{\text{i2t}} = {\text{b}}_{{{\text{Et}},{\text{t}} - 1}} {\text{E}}_{{{\text{i2t}} - 1}} + \beta_{\text{k}} {\text{y}}^{*}_{{{\text{i1t}} - 1}} + \alpha_{\text{k}} {\text{y}}^{*}_{{{\text{i2t}} - 1}} + \zeta_{\text{Ei2t}} , $$

where the Ph->E transmission parameters are α_k_ (transmission within a twin member) and β_k_ (transmission across twin members). By including the β_k_ parameter, we allow the phenotypic value of one twin member to contribute to the environment of the other twin member.

Application of the tracing rules reveals that Ph->E transmission starting at t = 1 necessarily changes the standard definition of the C and E at t = 2 and onwards, as the environmental influences become correlated. The tracing rules also reveal that the extension introduces GE covariance, i.e., the additive genetic variables (A; at t = 2,…,T), and the unshared environmental influences (E; at t = 2,…,T) are correlated. de Kort et al. (2012) considered this model, but accommodated C by allowing the environmental influences to be correlated (i.e., not modeling E and C, but T, the totality of relevant environmental influences; Carey, 2006). The model depicted in Fig. 2, and de Kort’s model are equivalent if b_Ct,t−1_ = b_Et,t−1_ (t = 2,…,T).

## Local Identification

We know that the standard genetic ACE simplex is locally identified, provided that the occasion-specific variances (σ^2^[a_t_], σ^2^[c_t_], and σ^2^[e_t_]) at t = 1 and t = T are fixed to zero, or constrained to equal the neighboring variances. Below we considered the identification of the extended model in two ways, numerically and analytically. A model is analytically locally identified if the Jacobian matrix of the model is of full column rank (Bekker et al. 1993). Let **σ**(**θ**) denote the (T*(T + 1)/2)-dimensional vector containing the non-redundant elements of the model covariance matrices of the MZ and DZ twins (expressed in terms of the fixed and to-be-estimated parameters), and let **θ** denote the p-dimensional vector of to-be-estimated parameters. The p × q Jacobian matrix equals J(**θ**) = ∂**σ**(**θ**)/∂**θ**. As explained by Bekker et al. (1993), the model is locally identified if J(**θ**) has full column rank. This test may be understood as a generalization of the test of the rank of the design matrix in scaling tests (Mather and Jinks 1977). We used Maple 6 (e.g., Heck 1993) to carry out this test by expressing in Maple the phenotypic covariance matrices in terms of the parameters, organizing these in the vector **σ**(**θ**), and defining the vector **θ**. This is a small programming task. The more complicated operations of calculating the Jacobian and its null space are carried out in Maple. For other of applications of this method in the twin design and in other contexts, see Derks et al. (2006), de Kort et al. (2012), and Bollen and Bauldry (2010).

Identification can also be established numerically by choosing parameters values, calculating the expected covariance matrices given these values, and establishing that the parameters are consistently (given variation in starting values) correctly recovered in fitting the true model to the expected covariance matrices. Analytical identification is computationally more demanding, but preferable as it does not depend on an arbitrary choice of parameter values. However, to reduce the computational burden in Maple, we imposed the following constraints on the occasion-specific variances: (1) $\sigma^{ 2} [{\text{a}}_{ 1} ] = \sigma^{ 2} [{\text{a}}_{ 2} ] = \sigma^{ 2} [{\text{a}}_{ 3} ] = \sigma^{ 2} [{\text{a}}_{ 4} ] \ne 0$, (2), σ^2^[c_t_]=0 (t=1,…,4), and (3) $\sigma^{ 2} [{\text{e}}_{ 1} ] = \sigma^{ 2} [{\text{e}}_{ 2} ] = \sigma^{ 2} [{\text{e}}_{ 3} ] = \sigma^{ 2} [{\text{e}}_{ 4} ] \ne 0$. In the numerical study of identification, we relax these constraints.

## Phenotype to E Effects in the Presence of C

### *Analytical Identification T* = *4*

Given T = 4, the addition of the parameters α_k_ and β_k_ (k = 1,2,3), rendered the extended simplex model (see Fig. 2) unidentified. In exploring identifying constraints, we first considered the parameters α_k_ and β_k_. Using Maple, we established that the reduction of two sets of three parameters (α_k_ and β_k_, k = 1,2,3) to two sets of two parameters rendered the model identified. We considered the equality constraints α_2_ = α_3_ and β_2_ = β_3_ (leaving α_1_ and β_1_ unconstrained), and we considered the imposition of a linear trend α_k_ = b_0α_ + (k−1)*b_1α_, k = 1,2,3, and β_k_ = b_0β_ + (k−1)*b_1β_ (k = 1,2,3), where b_0α_, b_1α_, b_0β_, and b_1β_ are now the free parameters. We note that whereas α_1_, α_2_ = α_3_ and β_1_,β_2_ = β_3_ is identified, we found that α_1_ = α_2_, α_3_ and β_1_ = β_2_, β_3_ is not. We repeated these analyses without common environmental influences (i.e., deleting the C simplex), but this had no bearing on the results, i.e., the equality constraints (or the linear constraints) were still required to render the model identified.

Others constraints are possible. An obvious choice is to constrain the autoregressive parameters. We considered separately b_A2,1_ = b_A3,2_ = b_A4,3_ (henceforth equal A b-coefficients), b_C2,1_ = b_C3,2_ = b_C4,3_ (equal C b-coefficients), and b_E2,1_ = b_E3,2_ = b_E4,3_ (equal E b-coefficients), and found that each set of constraints resulted in model identification with unconstrained α_k_ and β_k_ (k = 1,2,3).

### *Analytical Identification T* = *3*

We retained the constraints on the occasion-specific variance (at t = 1,2,3). The T = 3 model with the two sets of two parameter, e.g., α_1_, α_2_ and β_1_, β_2_, is not identified (deletion of the common environmental simplex did not results in identification). Imposing equality constraints on the parameters α_k_ and β_k_ (α_1_ = α_2_ = α and β_1_ = β_2_ = β) rendered the model identified. We then considered the imposition of (1) equal A b-coefficients and equal C b-coefficients; (2) equal A b-coefficients and equal E b-coefficient; (3) equal C b-coefficients and equal E b-coefficients. Each sets of constraints rendered the model identified, with unconstrained α_k_ and β_k_ (k = 1,2).

In sum, we considered identification subject to constraints on the occasion-specific residuals mentioned above. Given T = 4, we conclude the following: (1) The extended model (Fig. 2) with added parameters α_k_ and β_k_ is identified given constraints on α_k_ (e.g., α_1_, α_2_ = α_3_; or the linear constraint) and on β_k_ (e.g., β_1_, β_2_ = β_3_; or the linear constraint). These constraints are required in the presence or absence of the C simplex. (2) It is identified with α_k_ (k = 1,2,3) and β_k_ (k = 1,2,3) given equality constrained autoregressive coefficient, i.e., given equal A b-coefficients, equal C b-coefficients, *or* equal E b-coefficients). Given T = 3, we conclude the following: (1) The extended model is identified given the equality constraints α_1_ = α_2_ and β_1_ = β_2_. (2) It is identified with α_k_ (k = 1,2) and β_k_ (k = 1,2) given equal A b-coefficients and equal C b-coefficients, or equal A b-coefficients and E b-coefficients, or equal E b-coefficients and equal C b-coefficients.

## Misspecification and Power Given T = 4

Local identification is a necessary, but not a sufficient, condition for a model to be viable. We have to provide some indication of power to resolve the effects of interest (Martin et al. 1978). Given the fairly restrictive identification conditions associated with T = 3, we address these issues only in the case of T = 4. We do this by fitting the true model and misspecified models to the population matrices using exact normal data simulation (van der Sluis et al. 2008). We used Mplus 6.1 (Muthén and Muthén 2007) to fit the models using maximum likelihood (ML) estimation.

We consider only three sets of parameter values. The first set includes the following parameters of the standard genetic simplex (ACE; set 1):

$$ {\text{b}}_{{{\text{At}},{\text{t}} - 1}} = . 7,{\text{ b}}_{{{\text{Ct}},{\text{t}} - 1}} = . 9,{\text{ and b}}_{{{\text{Et}},{\text{t}} - 1}} = . 6, \quad \left( {{\text{t}} = 2, 3, 4} \right) $$

$$ \sigma^{ 2} [\zeta_{\text{A1}} ] = . 4*\rho ,\,\sigma^{ 2} [\zeta_{\text{C1}} ] = . 2*\rho ,\,\sigma^{ 2} [\zeta_{\text{E1}} ] = . 4*\rho , $$

$$ \sigma^{ 2} [\zeta_{\text{At}} ] = . 4*\left( { 1- {\text{ b}}_{{{\text{At}},{\text{t}} - 1}}^{ 2} } \right)*\rho , $$

$$ \sigma^{ 2} [\zeta_{\text{Ct}} ] = . 2*\left( { 1- {\text{ b}}_{{{\text{Ct}},{\text{t}} - 1}}^{ 2} } \right)*\rho , $$

$$ \sigma^{ 2} [\zeta_{\text{Et}} ] = . 4*\left( { 1- {\text{ b}}_{{{\text{Et}},{\text{t}} - 1}}^{ 2} } \right)*\rho , \quad \left( {{\text{t}} = 2, 3, 4} \right) $$

$$ \sigma^{ 2} [{\text{a}}_{\text{t}} ] = \left( { 1- \rho } \right)*. 50,\sigma^{ 2} [{\text{c}}_{\text{t}} ] = 0, \, \;{\text{and}}\;\;\sigma^{ 2} [{\text{e}}_{\text{t}} ] = \left( { 1- \rho } \right)*. 50. \quad \left( {{\text{t}} = 1,.., 4} \right) $$

The second set includes σ^2^[c_t_] as shown (ACE; set 2):

$$ {\text{b}}_{{{\text{At}},{\text{t}} - 1}} = . 7,{\text{ b}}_{{{\text{Ct}},{\text{t}} - 1}} = . 9,{\text{ and b}}_{{{\text{Et}},{\text{t}} - 1}} = . 6, \quad \left( {{\text{t}} = 1, 2, 3} \right) $$

$$ \sigma^{ 2} [\zeta_{\text{A1}} ] = . 4*\rho ,\,\sigma^{ 2} [\zeta_{\text{C1}} ] = . 2*\rho ,\,\sigma^{ 2} [\zeta_{\text{E1}} ] = . 4*\rho , $$

$$ \sigma^{ 2} [\zeta_{\text{At}} ] = . 4*\left( { 1- {\text{ b}}_{{{\text{At}},{\text{t}} - 1}}^{ 2} } \right)*\rho , $$

$$ \sigma^{ 2} [\zeta_{\text{Ct}} ] = . 2*\left( { 1- {\text{ b}}_{{{\text{Ct}},{\text{t}} - 1}}^{ 2} } \right)*\rho , $$

$$ \sigma^{ 2} [\zeta_{\text{Et}} ] = . 4*\left( { 1- {\text{ b}}_{{{\text{Et}},{\text{t}} - 1}}^{ 2} } \right)*\rho , \quad \left( {{\text{t}} = 2, 3, 4} \right) $$

$$ \sigma^{ 2} [{\text{a}}_{\text{t}} ] = \left( { 1- \rho } \right)*. 3 3 3,\;\;\sigma^{ 2} [{\text{c}}_{\text{t}} ] = . 3 3 3, \quad \;{\text{and}}\;\;\sigma^{ 2} [{\text{e}}_{\text{t}} ] = \left( { 1- \rho } \right)*. 3 3 3. \quad \left( {{\text{t}} = 1,.., 4} \right) $$

The third set excludes the influence of C altogether, i.e., b_Ct,t−1_ = .0, σ^2^[ζ_C1_] = 0, σ^2^[ζ_Ct_] = 0, σ^2^[c_t_] = 0 (AE; set 3):

$$ {\text{b}}_{{{\text{At}},{\text{t}} - 1}} = . 7,{\text{ and b}}_{{{\text{Et}},{\text{t}} - 1}} = . 6, \quad \left( {{\text{t}} = 2, 3, 4} \right) $$

$$ \sigma^{ 2} [\zeta_{\text{A1}} ] = . 5*\rho ,\;\;\sigma^{ 2} [\zeta_{\text{E1}} ] = . 5*\rho , $$

$$ \sigma^{2} [\zeta_{\text{At}} ] = .5*\left( {1 - {\text{b}}_{{{\text{At}},{\text{t}} - 1}}^{2} } \right)*\rho, \sigma^{2} [\zeta_{\text{Et}} ] = .5*\left( {1 - {\text{b}}_{{{\text{Et}},{\text{t}} - 1}}^{2} } \right)*\rho,\quad \left( {{\text{t}} = 2,3,4} \right) $$

$$ \sigma^{ 2} [{\text{a}}_{\text{t}} ] = \left( { 1- \rho } \right)*. 50,\;{\text{ and}}\;\;\sigma^{ 2} [{\text{e}}_{\text{t}} ] = \left( { 1- \rho } \right)*. 50. \quad \left( {{\text{t}} = 1,.., 4} \right) $$

Note that the fixed parameter ρ is the ratio of the variance due to the autoregressive processes to the total phenotypic variance (ρ is the reliability, if we conveniently consider the occasion-specific variance as due to *error*). We chose ρ = .80, so that 20 % of the phenotypic variance is occasion-specific in the standard simplex. Given three sets of parameters, we varied α_k_ and β_k_ as shown Table 1. In fitting the models, we consistently applied the identifying constraints α_2_ = α_3_ (α_1_ unconstrained) and β_2_ = β_3_ (β_1_ unconstrained). In set 1 and 3, we imposed the constraints mentioned on the occasion-specific residual variances: σ^2^[a_1_] = σ^2^[a_2_] = σ^2^[a_3_] = σ^2^[a_4_] ≠ 0, σ^2^[c_t_] = 0 (t = 1,…,4), and σ^2^[e_1_] = σ^2^[e_2_] = σ^2^[e_3_] = σ^2^[e_4_] ≠ 0. In set 2, we included the occasion-specific variances σ^2^[c_1_] = σ^2^[c_2_] = σ^2^[c_3_] = σ^2^[c_4_] ≠ 0 to establish numerically that these are identified.

**Table 1:** Detection of the phenotype to environment transmission in the ACE simplex and in the AE simplex

α_k_	β_k_	χ^2^	~N
ACE	(parameter set 1)		
.10	.10	2.096	11,700
.10	.15	5.034	4,700
.15	.10	1.976	12,100
.15	.15	4.885	4,800
−.10	.10	2.487	9,600
.10	−.10	5.334	4,500
−.10	−.10	3.670	6,500
ACE	(parameter set 2)		
.10	.10	1.234	19,300
.10	.15	2.588	9,250
.15	.10	1.283	18,650
.15	.15	2.735	8,750
−.10	.10	0.907	26,350
.10	−.10	6.649	3,600
−.10	−.10	3.892	6,100
AE	(parameter set 3)		
.10	.10	38.408	620
.10	.15	89.690	260
.15	.10	41.679	580
.15	.15	97.266	240
−.10	.10	27.754	860
.10	−.10	27.756	860
−.10	−.10	21.653	1,100

The χ^2^ equals the non-centrality parameter times N (N = NMZ (1000) + NDZ (1000)) obtained by fitting the model with the Ph->E transmission parameters (α_1_, α _2_ = α_3_, β_1_, β_2_ = β_3_) fixed to zero. The approximate sample size (~N) required is based on a power calculation given the type I error probability of alpha = 0.05 and df = 4, and an equal number of MZ and DZ twin pairs (N = Nmz + Ndz)

The parameter values chosen are quite arbitrary. To get some sense of the resulting summary statistics, we report in the appendix associated phenotypic summary statistics associated with parameter sets 1 and 3. The twin correlations look plausible. The models gives rise to small differences in phenotypic variance in the MZs and DZs (Eaves et al. 1977). In both set 1 and 3, given α_k_ = β_k_ = .1, the within twin member correlations between the As and Es range from .0 to .32 in MZs, and from .0 to .25 in DZs. Given α_k_ = β_k_ = −.1, the correlations range from −.07 to −.28 in MZs and from −.05 to −.21 in DZs. In sum, the results in Table 1 are based on the model with the (over-identifying) constraints on the occasion-specific residual variances, and on the constraints α_2_ = α_3_ (α_1_ unconstrained) and β_2_ = β_3_ (β_1_ unconstrained).

The results in Table 1 are clear: given the present parameter values, we require prohibitively large sample sizes to resolve Ph->E transmission in the presence of C. This is understandable as Ph->E transmission destroys a design feature of the ACE model: A, C, and E become correlated over time. The resolution in set 2 is slightly lower still, but the differences are relatively small (i.e., the resolution is dismal, regardless). It is important to note that the addition of the occasion-specific residual variances, σ^2^[c_t_], did not give rise to any identification problems, judging by the parameter recovery and the Mplus numerical identification test based on the Information matrix.

Ph->E transmission in the AE model, in contrast, fares well in terms of sample size requirements to resolve this feature (see Table 1). The results raise the question how well we can distinguish between the AE model with Ph->E transmission and the ACE model without such transmission. Although these models are not nested, we can still compare the overall χ^2^ goodness of fit indices, as obtained by fitting the models to the population covariance matrices. These results are shown in Table 2. The generating model is the AE simplex (parameter set 3) with the parameters α_1_, α_2_ = α_3_, β_1_, and β_2_ = β_3_. We fitted the AE simplex model (df = 68) without the parameters α_k_ = β_k_ = 0 (same results as in Table 1, set 3), we fitted the standard ACE models with and with occasion-specific residuals (df = 61 and df = 60, respectively), and the AE model without a rank one C covariance matrix (df = 64; i.e., we estimated σ^2^[C_1_] = σ^2^[ζ_C1_] and b_Ct,t−1_, but fixed σ^2^[ζ_Ct_] = 0, t = 2,3,4, and σ^2^[c_t_] = 0). Judging by the twin correlations in the Table 5 in Appendix (set 3), the inclusion of C makes little sense if β_k_ is negative. However, we proceed with the model fitting results, but return to the model α_k_ = .1 & β_k_ = −.1 below.

**Table 2:** The generating model is the AE simplex (parameter set 3) with the parameters α_1_, α_2_ = α_3_, β_1_, and β_2_ = β_3_, as shown

α_k_	β_k_	AE simplex	ACE simplex	ACE simplex	AE simplex C rank 1
		df = 68	df = 61	df = 60	df = 64
.10	.10	4.39 + 34.01	1.55 + 3.89	1.55 + 3.89	1.55 + 3.89
.10	.15	8.97 + 80.72	3.12 + 8.10	3.12 + 8.10	3.12 + 8.12
.15	.10	4.70 + 36.97	1.60 + 4.02	1.60 + 4.02	1.60 + 4.02
.15	.15	9.55 + 87.71	3.19 + 8.35	3.19 + 8.35	3.19 + 8.35
−.10	.10	3.40 + 24.35	1.45 + 3.86	1.45 + 3.86	1.45 + 3.86
.10	−.10	6.15 + 21.61	5.71 + 18.45	5.71 + 18.45	5.84 + 19.30
−.10	−.10	4.52 + 17.14	3.94 + 14.04	3.95 + 14.05	4.13 + 14.55

The χ^2^ goodness of fit indices are associated with the incorrect models: the AE simplex, the ACE simplex, and the AE simplex with rank 1 C (i.e., σ^2^[ζ_Ct_] = 0, t = 2,3,4). In these models the parameters α_k_ and β_k_ (k = 1,2,3) were fixed to zero. The total χ^2^, given NMZ = NDZ = 1000, is broken down into the MZ and the DZ contributions, respectively

The 68 df model is the standard AE simplex with occasion-specific variances σ^2^[a_t_] = 0 & σ^2^[e_t_] = 0. This model is nested under the true model (i.e., AE simplex with Ph->E transmission parameters α_k_ and β_k_). These results are also given in Table 1

The 61 df model is the standard ACE simplex with occasions specific variances σ^2^[a_t_], σ^2^[e_t_], and σ^2^[c_t_]

The 60 df model is the standard ACE simplex with occasions specific variances σ^2^[a_t_], σ^2^[e_t_], and σ^2^[c_t_] = 0

The 64 df model is the standard AE simplex with occasions specific variances σ^2^[a_t_], σ^2^[e_t_], and σ^2^[c_t_] = 0, and a rank one C covariance matrix (i.e., σ^2^[ζ_Ct_] = 0, t = 2,3,4)

The goodness of fit results (df = 61 model vs. df = 60 models) are consistent with our finding that the occasion-specific residuals variances consistently hit the lower bound of zero (σ^2^[c_t_] = 0), as did the C innovation variances (i.e., σ^2^[ζ_Ct_] = 0, t = 2,3,4). Dropping these variance components did not result in any appreciable increase in χ^2^ (the df = 64 model). The results in Table 2 suggest that (1) the power is good to detect the Ph->E transmission in the AE model (as we know from Table 1; e.g., given α_k_ = β_k_ = .1, chi2 = 4.39 + 34.01 = 38.4, df = 4); (2) the ACE model will fit the AE model with Ph->E transmission quite well as long as β_k_ is positive (e.g., given α_k_ = β_k_ = .1, χ^2^ = 1.55 + 3.89 = 5.44; DF = 64; Nmz = Ndz = 1000); (3) the C in the misspecified ACE model is almost perfectly rank 1 (compare column 2 and 3 of Table 2); (4) the DZ twins generally provide most information to distinguish these models.

To show that the incorrect df = 64 model (AE simplex, C rank 1) not only fits well, but also produces seemingly sensible parameter values, we report the parameters of this incorrect model, given the data generating AE simplex model with α_k_ = .15 and β_k_ = .15. The point estimates (standard errors in parentheses) are:

$$ \sigma [{\text{a}}_{\text{t}} ] = . 2 8 6 { }\left( {.0 4} \right),\quad \sigma [{\text{e}}_{\text{t}} ] = . 3 1 8 { }\left( {.0 4} \right), $$

$$ \sigma [\zeta_{\text{A1}} ] = . 5 2 8 { }\left( {.0 4} \right),\quad \sigma [\zeta_{\text{A2}} ] = . 3 9 4 { }\left( {.0 6} \right),\quad \sigma [\zeta_{\text{A3}} ] = . 3 9 1 { }\left( {.0 6} \right),\quad \sigma [\zeta_{\text{A4}} ] = . 4 2 4 { }\left( {.0 6} \right), $$

$$ \sigma [\zeta_{\text{E1}} ] = . 6 4 7 { }\left( {.0 2} \right),\quad \sigma [\zeta_{\text{E2}} ] = . 5 1 2 { }\left( {.0 3} \right),\quad \sigma [\zeta_{\text{E3}} ] = . 5 1 2 { }\left( {.0 3} \right),\quad \sigma [\zeta_{\text{E4}} ] = . 50 9 { }\left( {.0 3} \right), $$

$$ \sigma [\zeta_{\text{C1}} ] = . 3 4 7 { }\left( {.0 4} \right), $$

$$ {\text{b}}_{{{\text{A2}}, 1}} = . 9 1 1 { }\left( {. 1 1} \right),{\text{ b}}_{{{\text{A3}}, 2}} = . 7 90 \, \left( {.0 8} \right),{\text{ b}}_{{{\text{A4}}, 3}} = . 7 8 3 { }\left( {.0 9} \right), $$

$$ {\text{b}}_{{{\text{E2}}, 1}} = . 5 8 2 { }\left( {.0 4} \right),{\text{ b}}_{{{\text{E3}}, 2}} = . 5 8 6 { }\left( {.0 4} \right),{\text{ b}}_{{{\text{E4}}, 3}} = . 5 8 9 { }\left( {.0 4} \right), $$

$$ {\text{b}}_{{{\text{C2}}, 1}} = 1. 5 6 { }\left( {. 1 3} \right),{\text{ b}}_{{{\text{C3}}, 2}} = 1. 3 3 { }\left( {.0 9} \right),{\text{ b}}_{{{\text{C4}}, 3}} = 1. 1 5 2 { }\left( {.0 7} \right). $$

These values seem quite sensible. One may object to the estimates of b_Ct+1,t_ being greater than one (the parameters being outside the unit circle). However, in this model these parameters are not interpretable as autoregressive coefficients.

We note that the ACE simplex model does not fit quite as well if the parameter β_k_ is negative (i.e., β_k_ = −.10). The summary statistics in the Appendix indicate that parameter set 3, with α_k_ = .10 and β_k_ = −.10, produces correlations that are not consistent with the presence of C. For instance, given α_k_ = .10 and β_k_ = −.10, we have, at t = 2,3,4, MZ correlations of .420, .373, and .345, and DZ correlations of .148, .090, and .054. These resemble twin correlations sometimes observed in personality dimensions. It is well known that negative sibling interaction and non-additive genetic effects may give rise to such disparate correlations (Rietveld et al. 2003a, b; Eaves 1988, 1976). Given that C is unlikely given these correlations, we fitted the standard ADE simplex to the data generated by set 3 with α_k_ = .10 and β_k_ = −.10. We obtained a χ^2^(60) of 14.37. As the occasion-specific D variances and the D innovation variances were zero, so we fixed these to zero (the D covariance matrix is now rank 1), and again obtained the χ^2^(64) = 14.37. The parameter values were quite sensible. One may question whether dominance is enough to account for the differences in the correlations (e.g., .345 vs. .054; Eaves, 1988). However, as above, what is puzzling, if one were to take the ADE model seriously, is the rank 1 D covariance matrix.

Finally, we considered the fit of the AE model with Ph->E transmission to expected covariance matrices generated with parameter set 2 (ACE simplex with α_k_ = β_k_ = 0). In Mplus the χ^2^(64) was 45.7 (Nmz = Ndz = 1000), which is relatively large compared to the values in Table 2. More importantly, we note that the parameter estimates made little sense. For instance, the parameters b_Et+1,t_ assumed negative values, and the parameters b_A2,1_ and b_A3,2_ were almost zero. In conclusion, we find that the AE model with Ph->E transmission fits data generated by the standard ACE simplex relatively poorly, and does not produce sensible parameter estimates.

## Illustration: Anxiety at 3, 7, 10, and 12

We applied the ACE standard simplex and the AE simplex with Ph->E transmission to mother ratings of anxiety measured at ages 3, 7, 10, and 12 years in female MZ and DZ twins. The data were collected by the Netherlands Twin Register (NT), which includes the Young NTR (YNTR; van Beijsterveldt et al. 2003; Boomsma et al. 2002, 2006) that has recruited newborn twins and multiples at birth since 1987. The parents and teachers of the twins rate anxious depression in the children by age appropriate questionnaires from the Achenbach system of empirical assessment (ASEBA): the Child Behavior Checklist (CBCL/1.5-5; Achenbach 1990, 1992a, b) and CBCL/4-18 (Verhulst et al. 1996).

As Ph->E transmission need not be the same in boys and girls, and as a proper treatment of sex differences is beyond the present scope, we analyzed the data of the MZ and DZ girls. We have 3,480 MZ pairs and 3,145 DZ pairs. The percentages observed at ages 3–12 years are about 89, 54, 45, and 37 % in MZ twins, and 89, 50, 39, and 32 % in the DZ twins. FIML estimates of the MZ phenotypic twin correlations are .71, .58, .58, and .63. The corresponding DZ correlations are .31, .36, .35, and .40. Additional summary statistics are given in Table 6 Appendix. Using FIML estimation in Mplus, we fitted to the raw data the standard ACE simplex model, with occasion-specific residual variances constrained to be equal over time.

The goodness of fit indices are shown in Table 3. We found that the occasions specific residual variances, σ[c_t_] (t = 1,…,4), and the C innovations were zero, σ[ζ_Ct_] (t = 2,3,4). We fixed these to zero, reducing the C covariance matrix to rank 1. We know from the analyses of expected covariance matrices (see above, Table 2) that the rank 1 C covariance matrix is compatible with Ph->E transmission. We therefore removed C altogether by dropping the parameter σ[ζ_C1_], and we added the four Ph->E transmission parameters α_1_, α_2_ = α_3_, β_1_, and β_2_ = β_3_. This resulted in smaller AIC and BIC, but the α_k_ parameters were not significant (alpha = .01). The model with only α_k_ = 0 and β_k_ (2 parameters) estimated produced the smallest value of BIC and a slightly larger AIC. As a check, we fitted the model with α_k_ estimated (2 parameters) and β_k_ = 0, but concluded that this model is not compatible with the data, as it consistently failed to converge. Finally we fitted the standard AE simplex. But this model produced the largest AIC and the third largest BIC. Given the values of α_k_ = 0, we conclude that a twin’s anxious behavior does not influence her own environment, but does contribute to the environment of her co-twin. We report in Table 4 the parameter estimates and robust standard errors.

**Table 3:** Fit indices (smallest AIC and BIC underlined)

	logl	npar	AIC	BIC
ACE standard simplex	−69528.5	28	139113	139,303
AE simplex C rank 1	−69528.5	24	139,105	139,268
AE simplex + α_k_, β_k_	−69519.3	24	139,086	139,249
AE simplex + β_k_	−69522.1	22	139,088	139,237
AE standard simplex	−69537.9	20	139,115	139,251

**Table 4:** Parameter estimates in the analysis of Anxiety from 3y to 12y. ML estimates and robust standard errors in parentheses in the AE simplex with parameter β_k_, logl = −69522.1)

	t = 1 (3y)	t = 2 (7y)	t = 3 (10y)	t = 4 (12y)
b_At,t−1_	–	0.277 (.062)	0.886 (.099)	0.790 (.100)
σ[ζ_At_]	2.450 (.098)	1.881 (.152)	1.189 (.302)	1.214 (.277)
b_Et,t−1_	–	0.414 (.182)	1.017 (.232)	0.799 (.125)
σ[ζ_Et_]	1.034 (.240)	1.173 (.229)	1.212 (.307)	0.728 (.477)
σ[a_t_]	0.956 (.227)	0.956 (.227)	0.956 (.227)	0.956 (.227)
σ[e_t_]	1.375 (.173)	1.375 (.173)	1.375 (.173)	1.375 (.173)
β_1_	0.123 (.041)			
β_2_ = β_3_	0.062 (.027)			

Table 7 in Appendix contains the correlation matrices among the A and E variables. We note that we observed correlations between the A and E variables after age 3, i.e., GE covariance attributable to the Ph->E transmission. The parameters estimates are β_1_ = 0.123 (s.e. .041) and β_2_ = β_3_ = 0.062 (s.e. .027), and the resulting correlations between A and E range from .05 to .23. From age 3 onwards, we note that the environmental variables become correlated, again due to the Ph->E transmission positive parameters β_k_. The correlations range from .09 to .23. Both the additive genetic correlations (.45 (3–7y), .87 (7–10y), .87 (10–12y)) and environmental correlations increase over time (.33, .74, and .89). The heritabilities are 0.70 (3y), 0.58 (7y), 0.51 (10y), 0.53 (12y). Given the positive values of the transmission parameters, we may interpret the results in the spirit of cooperative sibling interaction (Eaves 1976; Eaves et al. 1977): manifest anxious behavior of one twin member forms a cause of anxiety in the other twin member, by contributing to the other twin’s environment.

## Discussion

We perceive a discrepancy between the practice of longitudinal modeling within the classical twin design and developmental psychological theory. The former usually features the provisional assumption that GE covariance is absent, while the latter places great emphasis on GE covariance arising in plausible notions of genotype–environment interplay or person–environment interplay (Loehlin and DeFries 1987; Plomin et al. 1977; Scarr 1992; Scarr and McCartney 1983). For instance, in developmental psychopathology, GE covariance is accorded an important role (Rutter et al. 1997, 2006; Rutter and Silberg 2002), and is thought to be relevant to the development of treatment (Jaffee and Price 2008). GE-covariance is also thought to be relevant to cognitive development (Johnson et al. 2011; Dickens and Flynn 2001; Haworth et al. 2010; for a recent application of the present model to intelligence data, see Dolan et al. 2014).

**Fig. 3:**
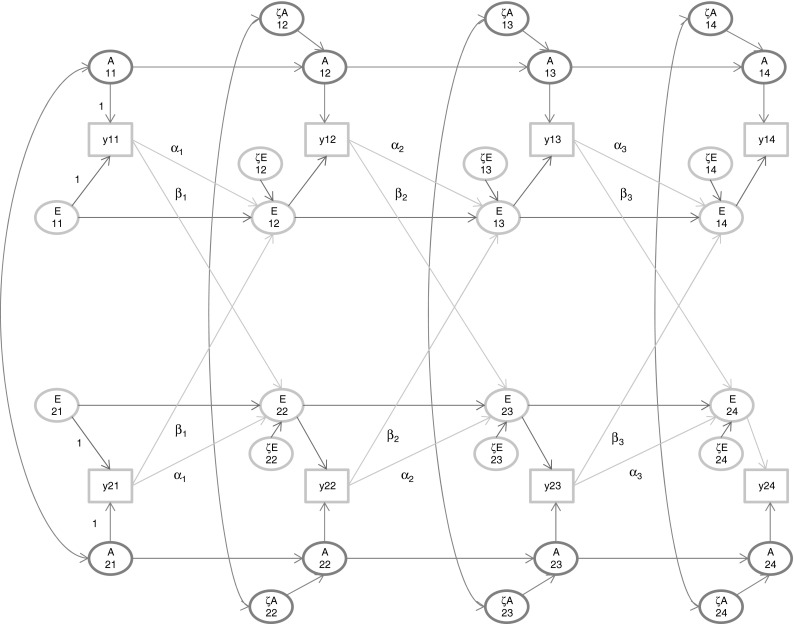
The extended AE simplex model. Occasion-specific influences are not shown. The scaling used is shown only at t = 1. The extension comprises the arrows from the phenotype y^*^ at t to the E variables at t + 1 (i.e., parameters α_k_ and β_k_). For the distinction between y (Fig. 1) and y^*^ in this Figure, see the text

In the present paper, we explored, in the longitudinal classical twin design, GE-covariance by positing Ph->E transmission, as discussed by Eaves (1976), Eaves et al. (1977), Carey (1986), and de Kort et al. (2012). Local identification of the model considered posed no great problems. Given T = 4, we established local identification given the constraints reducing the two set of three parameters (α_k_, β_k_; k = 1,2,3) to sets of two parameters (α_2_ = α_3_ and β_2_ = β_3_ or a linear constraint), in otherwise unconstrained genetic and environmental simplex models. The parameters α_k_, β_k_ (k = 1,2,3) may be rendered identified by introducing other constraints in the simplex (equal autoregressive coefficients), but we did not pursue such constraints in our numerical analyses. Our numerical results—given our limited choice of parameters—suggest that Ph->E transmission in the absence of C is viable with realistic samples sizes (Fig. 3), but, in the presence of C (Fig. 2), well beyond the resolution provided by realistic twin samples in the presence of C (see Table 1). This is understandable, as Ph->E transmission (without explicit C influences) gives rise to correlated environmental effects, which are hard to distinguish from proper C (see Table 6 in Appendix).

An interesting result is that the AE simplex Ph->E transmission, with positive α_k_ and β_k_, gives rise to a covariance structure that is quite consistent with an AE simplex plus a rank 1 C covariance matrix. The presence of C is to be expected, given the correlated environmental effects caused by the Ph->E transmission. However, we had not anticipated that the resulting C covariance matrix is rank 1. We contend that a rank 1 covariance matrix (be it due to A, D, C, or E) is in itself a suspicious results (what psychological process generates this?). It is striking that we also observed the rank 1 C covariance matrix in our analyses of the anxiety data. We found the Ph->E transmission model, limited to β_k_, provided the best fit in terms of the AIC and BIC. In addition, compared to the model with a rank 1 C covariance matrix, we think that this model is substantively more plausible. GE-covariance, as conceived here, necessarily gives rise to correlated environments. So a successful AE model would seem to rule out our GE covariance process. However, we note that an ADE model, notably with a rank 1 D covariance matrix, is compatible with Ph->E transmission with a negative parameter β_k_. A second symptom of GE covariance in this connection is an overly large discrepancy between the phenotypic MZ and the DZ correlation (e.g., as mentioned above, .345 vs. .054; but see Eaves 1988, for a genetic explanation).

We have considered only Ph->E transmission (Fig. 3), but recognize that there are other possibilities. We considered phenotype to E (within twin member; behavior influences own environment, E) in combination with phenotype to C transmission (Ph->C). This is formally locally identified given constraints to those applied to α_k_ and β_k_. However, numerically this model generated many problems, which suggested empirical under-identification. We did not consider phenotype to A transmission, although we consider it possible that behavior (e.g., substance abuse, exercise, etc.) may influences gene expression. We do not know whether such feedback is detectable in psychometric data, or on the time scale upon which such data are typically collected. In addition, we do not know how well we can distinguish statistically these various types of feedback models. This question is relevant to our analyses of anxiety. Eaves et al. (1986) interpreted phenotype to phenotype transmission as a test–retest effect. This could apply to the mothers’ ratings, although our results favored our model with phenotype to E. The model with phenotype to phenotype transmission within and between twin members produced a log likelihood of −69,522 (24 parameters), AIC = 139,093, and BIC = 139,256. The model with phenotype to phenotype transmission within twin members produced a log likelihood of −69,537 (22 parameters), AIC = 139,119, and BIC = 139,268. As shown in Table 3, AIC and BIC of our model of choice (22 parameters) equal 139,088 and 139,237, respectively.

Finally, we considered GE covariance in the absence of GE interaction. One form of interaction which would seem to be plausible is differential Ph->E transmission, in which the magnitude of the transmission effects depends on the phenotypic scores. For instance, in theories of cognitive development active Ph->E transmission is often associated with the idea of highly intelligent children seeking out (or creating) “smart” environments (Plomin et al. 1977). This may be true, but it does not necessarily imply that children of intermediate or low intelligence do not engage in any “niche” picking. Moderated Ph->E transmission may be modeled using Bayesian estimation (Eaves and Erkanli 2003).

## Appendix

See Tables 5, 6 and 7.

**Table 5:** Twin correlations and phenotypic variances associated with parameter sets 1 and 3

α	β	t=	Phenotypic variance				Twin correlations			
			1	2	3	4	1	2	3	4
Set 1										
0	0	mz	1	1	1	1	.580	.580	.580	.580
		dz	1	1	1	1	.370	.370	.370	.370
.1	.1	mz	1	1.211	1.367	1.481	.580	.653	.692	.716
		dz	1	1.185	1.323	1.424	.370	.468	.524	.557
.1	.15	mz	1	1.262	1.484	1.660	.580	.681	.733	.763
		dz	1	1.224	1.417	1.577	.370	.509	.582	.627
.15	.1	mz	1	1.282	1.510	1.694	.580	.656	.703	.733
		dz	1	1.254	1.460	1.624	.370	.472	.537	.580
.15	.15	mz	1	1.336	1.643	1.919	.580	.685	.744	.781
		dz	1	1.295	1.566	1.811	.370	.513	.597	.652
−.10	.10	mz	1	0.968	0.962	0.962	.580	.632	.640	.642
		dz	1	0.948	0.939	0.938	.370	.443	.457	.460
.10	−.10	mz	1	1.044	1.073	1.091	.580	.512	.471	.447
		dz	1	1.070	1.117	1.148	.370	.279	.226	.193
−.10	−.10	mz	1	0.840	0.803	0.794	.580	.500	.477	.471
		dz	1	0.859	0.827	0.818	.370	.266	.238	.230
Set 3										
0	0	mz	1	1	1	1	.500	.500	.500	.500
		dz	1	1	1	1	.250	.250	.250	.250
.1	.1	mz	1	1.184	1.310	1.396	.500	.577	.618	.642
		dz	1	1.152	1.256	1.326	.250	.348	.402	.434
.1	.15	mz	1	1.226	1.406	1.544	.500	.610	.665	.697
		dz	1	1.178	1.322	1.435	.250	.394	.469	.513
.15	.1	mz	1	1.250	1.438	1.580	.500	.580	.627	.658
		dz	1	1.216	1.375	1.493	.250	.350	.413	.454
.15	.15	mz	1	1.294	1.547	1.761	.500	.613	.676	.716
		dz	1	1.243	1.451	1.626	.250	.396	.483	.539
−.10	.10	mz	1	0.960	0.953	0.952	.500	.562	.572	.574
		dz	1	0.936	0.924	0.922	.250	.335	.351	.355
.10	−.10	mz	1	1.056	1.091	1.114	.500	.420	.373	.345
		dz	1	1.088	1.146	1.185	.250	.148	.090	.054
−.10	−.10	mz	1	0.864	0.837	0.832	.500	.421	.403	.399
		dz	1	0.888	0.866	0.862	.250	.155	.134	.130

**Table 6:** FIML estimates of summary statistics

	MZ GIRL 1				MZ GIRL 2			
	ANX3	ANX7	ANX10	ANX12	ANX3	ANX7	ANX10	ANX12
Mean:	3.812	2.325	2.696	2.437	3.688	2.211	2.570	2.365
Var:	9.900	7.491	10.78	10.71	10.15	7.518	10.84	10.11
Cor:	1.000							
	0.306	1.000						
	0.246	0.557	1.000					
	0.255	0.531	0.672	1.000				
	0.708	0.242	0.231	0.204	1.000			
	0.278	0.578	0.403	0.379	0.271	1.000		
	0.202	0.406	0.577	0.456	0.261	0.542	1.000	
	0.232	0.447	0.473	0.633	0.231	0.485	0.642	1.000
Mean:	3.775	2.722	2.803	2.623	3.492	2.281	2.613	2.219
Var:	9.940	11.27	11.80	11.89	9.355	8.458	11.31	9.490
Cor:	1.000							
	0.298	1.000						
	0.229	0.611	1.000					
	0.235	0.506	0.605	1.000				
	0.316	0.197	0.198	0.207	1.000			
	0.212	0.360	0.285	0.245	0.289	1.000		
	0.178	0.287	0.353	0.297	0.196	0.596	1.000	
	0.205	0.269	0.267	0.404	0.222	0.425	0.618	1.000

**Table 7:** Derived summary statistics (AE simplex + β_k_)

	A1				E1				A2				E2			
	3y	7y	10y	12y	3y	7y	10y	12y	3y	7y	10y	12y	3y	7y	10y	12y
MZ girls Correlations																
A1	1.00															
	0.34	1.00														
	0.28	0.83	1.00													
	0.23	0.67	0.81	1.00												
E1	0.00	0.00	0.00	0.00	1.00											
	0.23	0.08	0.07	0.05	0.33	1.00										
	0.20	0.13	0.11	0.09	0.25	0.74	1.00									
	0.21	0.19	0.18	0.15	0.22	0.66	0.89	1.00								
A2	1.00	0.34	0.28	0.23	0.00	0.23	0.20	0.21	1.00							
	0.34	1.00	0.83	0.67	0.00	0.08	0.13	0.19	0.34	1.00						
	0.28	0.83	1.00	0.81	0.00	0.07	0.11	0.18	0.28	0.83	1.00					
	0.23	0.67	0.81	1.00	0.00	0.05	0.09	0.15	0.23	0.67	0.81	1.00				
E2	0.00	0.00	0.00	0.00	0.00	0.10	0.09	0.09	0.00	0.00	0.00	0.00	1.00			
	0.23	0.08	0.07	0.05	0.10	0.12	0.14	0.17	0.23	0.08	0.07	0.05	0.33	1.00		
	0.20	0.13	0.11	0.09	0.09	0.14	0.14	0.20	0.20	0.13	0.11	0.09	0.25	0.74	1.00	
	0.21	0.19	0.18	0.15	0.09	0.17	0.20	0.25	0.21	0.19	0.18	0.15	0.22	0.66	0.89	1.00
Variances																
	A1				E1				A2				E2			
	6.00	4.00	4.55	4.32	1.07	1.67	3.27	2.74	6.00	4.00	4.55	4.32	1.07	1.67	3.27	2.74
Occasion-specific variances																
	0.91	0.91	0.91	0.91	1.89	1.89	1.89	1.89	0.91	0.91	0.91	0.91	1.89	1.89	1.89	1.89
DZ girls																
Correlations																
A1	1.00															
	0.34	1.00														
	0.28	0.83	1.00													
	0.23	0.67	0.81	1.00												
E1	0.00	0.00	0.00	0.00	1.00											
	0.12	0.04	0.03	0.03	0.33	1.00										
	0.11	0.07	0.06	0.04	0.25	0.74	1.00									
	0.12	0.10	0.10	0.08	0.22	0.66	0.89	1.00								
A2	0.50	0.17	0.14	0.11	0.00	0.23	0.20	0.20	1.00							
	0.17	0.50	0.42	0.34	0.00	0.08	0.13	0.18	0.34	1.00						
	0.14	0.42	0.50	0.41	0.00	0.07	0.11	0.18	0.28	0.83	1.00					
	0.11	0.34	0.41	0.50	0.00	0.05	0.09	0.14	0.23	0.67	0.81	1.00				
E2	0.00	0.00	0.00	0.00	0.00	0.10	0.09	0.09	0.00	0.00	0.00	0.00	1.00			
	0.23	0.08	0.07	0.05	0.10	0.09	0.11	0.15	0.12	0.04	0.03	0.03	0.33	1.00		
	0.20	0.13	0.11	0.09	0.09	0.11	0.12	0.18	0.11	0.07	0.06	0.04	0.25	0.74	1.00	
	0.20	0.18	0.18	0.14	0.09	0.15	0.18	0.22	0.12	0.10	0.10	0.08	0.22	0.66	0.89	1.00
Variances																
	A1				E1				A2				E2			
	6.00	4.00	4.55	4.32	1.07	1.67	3.26	2.72	6.00	4.00	4.55	4.32	1.07	1.67	3.26	2.72
Occasion-specific variances																
	0.91	0.91	0.91	0.91	1.89	1.89	1.89	1.89	0.91	0.91	0.91	0.91	1.89	1.89	1.89	1.89

These results can be understood by applying the path diagram tracing rules to Fig. 3 (bearing in mind α_k_ = 0). Specifically the MZ A1-E1 are half the DZ A1-E1 correlations, because, in tracing from (say) A1-3y to E1-7y, the additive genetic correlation is included in the route. The MZ and DZ A1-E2 correlations do not differ greatly because these are dominated by the β_k_ path, which connect the A of one twin (say A1-10y) with the E of the other twin (E2-12y) via the former’s phenotype. There is another indirect route that does involve the genetic correlation. However, this route so circuitous that its contribution is small

The underlined values are G-E correlations

## References

[CR1] Achenbach TM (1990). The Young Adult Self Report. Burlington, VT: University of Vermont, Dept. of Psychiatry; 1990

[CR2] Achenbach TM (1992). Manual for the child behavior checklist/2-3 and 1992 profile.

[CR3] Achenbach TM (1992). Integrative guide for the 1991 CBCL 4/18, YSR, and TRF profiles.

[CR4] Bartels M, Rietveld MJH, van Baal GCM, Boomsma DI (2002). Genetic and environmental influences on the development of intelligence. Behav Genet.

[CR5] Bartels M, van den Oord EJ, Hudziak JJ, Rietveld MJ, van Beijsterveldt CEM, Boomsma DI (2004). Genetic and environmental mechanisms underlying stability and change in problem behaviors at ages 3, 7, 10, and 12. Dev Psychol.

[CR6] Bekker PA, Merkens A, Wansbeek TJ (1993). Identification, equivalent models, and computer algebra.

[CR7] Bollen KA, Bauldry S (2010). Model identification and computer algebra. Sociol Methods Res.

[CR8] Boomsma DI, Molenaar PCM (1987). The genetic analysis of repeated measures. I. Simplex models. Behav Genet.

[CR9] Boomsma DI, Martin NG, Molenaar PCM (1989). Factor and simplex models for repeated measures: application to two psychomotor measures of alcohol sensitivity in twins. Behav Genet.

[CR10] Boomsma DI, Vink JM, van Beijsterveldt TC, de Geus EJ, Beem AL, Mulder EJ, Derks EM, Riese H, Willemsen GA, Bartels M, van den Berg M, Kupper NH, Polderman TJ, Posthuma D, Rietveld MJ, Stubbe JH, Knol LI, Stroet T, van Baal GC (2002). Netherlands twin register: a focus on longitudinal research. Twin Res.

[CR11] Boomsma DI, de Geus EJ, Vink JM (2006). Netherlands Twin Register: from twins to twin families. Twin Res Hum Genet.

[CR12] Cardon LR, Fulker DW, DeFries JC (1992). Continuity and change in general cognitive ability from 1 to 7 years of age. Dev Psychol.

[CR13] Carey G (1986). Sibling imitation and contrast effects. Behav Genet.

[CR14] de Kort JM, Dolan CV, Boomsma DI (2012). Accommodation of GE-covariance in a longitudinal twin design. Neth J Psychol.

[CR15] Derks EM, Dolan CV, Boomsma DI (2006). A test of the equal environment assumption (EEA) in multivariate twin studies. Twin Res Hum Genet.

[CR16] Dickens WT, Flynn JR (2001). Heritability estimates versus large environmental effects: the IQ paradox resolved. Psychol Rev.

[CR17] Dolan CV, Molenaar PCM, Boomsma DI (1991). Simultaneous genetic analysis of longitudinal means and covariance structure in the simplex model using twin data. Behav Genet.

[CR18] Dolan CV, de Kort JM, Kan K-J, van Beijsterveldt CEM, Bartels M, Boomsma DI (2014) Can GE-covariance originating in phenotype to environment transmission account for the Flynn effect? J Intell 10.1007/s10519-014-9659-5 PMC4023080 24789102

[CR19] Eaves LJ (1976). A model for sibling effects in man. Heredity.

[CR20] Eaves LJ (1988). Domiance alone is not enough. Behav Genet.

[CR21] Eaves LJ, Erkanli A (2003). Markov Chain Monte Carlo approaches to analysis of genetic and environmental components of human developmental change and G X E interaction. Behav Genet.

[CR22] Eaves LJ, Last K, Martin NG, Jinks JL (1977). A progressive approach to non-additivity and genotype-environmental covariance in the analysis of human differences. Br J Math Stat Psychol.

[CR23] Eaves LJ, Long J, Heath AC (1986). A theory of developmental change in quantitative phenotypes applied to cognitive development. Behav Genet.

[CR24] Gillespie NA, Evans D, Wright MJ, Martin NG (2007). Genetic simplex modeling of the dimensions of adolescent personality in a sample of young Australian twins. Twin Res.

[CR25] Haworth CMA, Wright MJ, Luciano M, Martin NG, de Geus EJ, van Beijsterveldt CEM (2010). The heritability of general cognitive ability increases linearly from childhood to young adulthood. Mol Psychiatr.

[CR26] Heck A (1993). Introduction to Maple, a computer algebra system.

[CR27] Hewitt JK, Eaves LJ, Neale MC, Meyer JM (1988). Resolving causes of developmental continuity or “tracking.” I. Longitudinal twin studies during growth. Behav Genet.

[CR28] Hoekstra RA, Bartels M, Boomsma DI (2007). Longitudinal genetic study of verbal and nonverbal IQ from early childhood to young adulthood. Learn Individ Differ.

[CR29] Jaffee SR, Price TS (2008). Gene–environment correlations: a review of the evidence and implications for prevention of mental illness. Mol Psychiatr.

[CR30] Johnson W, Penke L, Spinath FM (2011). Heritability in the era of molecular genetics: some thoughts for understanding. Eur J Pers.

[CR31] Jöreskog KG (1970). Estimation and testing of simplex models. Br J Math Stat Psychol.

[CR32] Loehlin JC, DeFries JC (1987). Genotype-environment correlation and IQ. Behav Genet.

[CR33] Martin NG, Eaves LJ, Kersey MJ, Davies P (1978). The power of the classical twin study. Heredity.

[CR34] Mather K, Jinks JL (1977). Introduction to biometrical genetics.

[CR35] Minica CC, Boomsma DI, van der Sluis S, Dolan CV (2010). Genetic association in multivariate phenotypic data: power in five models. Twin Res Hum Genet.

[CR36] Muthén LK, Muthén BO (2007). Mplus user’s guide.

[CR37] Petrill SA, Hewitt JK, Cherny SS, Lipton PA, Plomin R, Corley R, DeFries JC (2004). Genetic and environmental contributions to general cognitive ability through the first 16 years of life. Dev Psychol.

[CR38] Plomin R, DeFries JC, Loehlin JC (1977). Genotype-environment interaction and correlation in the analysis of human behavior. Psychol Bull.

[CR39] Rietveld MJH, Dolan CV, van Baal GCM, Boomsma DI (2003). A twin study of differentiation of cognitive abilities in childhood. Behav Genet.

[CR40] Rietveld MJH, Posthuma D, Dolan CV, Boomsma DI (2003). ADHD: sibling interaction or dominance: an evaluation of statistical power. Behav Genet.

[CR42] Rutter M, Silberg J (2002). Gene–environment interplay in relation to emotional and behavioral disturbance. Annu Rev Psychol.

[CR43] Rutter M, Dunn J, Plomin R, Simonoff E, Pickles A, Maughan B, Ormel J, Meyer J, Eaves L (1997). Integrating nature and nurture: implications of person–environment correlations and interactions for developmental psychopathology. Dev Psychopathol.

[CR44] Rutter M, Moffitt TE, Caspi A (2006). Gene–environment interplay and psychopathology: multiple varieties but real effects. J Child Psychol Psychiatry.

[CR45] Scarr S (1992). Developmental theories for the 1990s: development and Individual differences. Child Dev.

[CR46] Scarr S, McCartney K (1983). How people make their own environments: a theory of genotype → environment effects. Child Dev.

[CR47] van Beijsterveldt CEM, Bartels M, Hudziak JJ, Boomsma DI (2003). Causes of stability of aggression from early childhood to adolescence: a longitudinal genetic analysis in Dutch twins. Behav Genetics.

[CR48] van der Sluis S, Dolan CV, Neale MC, Posthuma D (2008). Power calculations using exact data simulation: a useful tool for genetic study designs. Behav Genet.

[CR49] Verhulst FC, van der Ende J, Koot HM (1996). Handleiding voor de CBCL/4-18 [Dutch manual for the CBCL/4-18].

